# Solution Scattering and FRET Studies on Nucleosomes Reveal DNA Unwrapping Effects of H3 and H4 Tail Removal

**DOI:** 10.1371/journal.pone.0078587

**Published:** 2013-11-12

**Authors:** Kurt Andresen, Isabel Jimenez-Useche, Steven C. Howell, Chongli Yuan, Xiangyun Qiu

**Affiliations:** 1 Department of Physics, Gettysburg College, Gettysburg, Pennsylvania, United States of America; 2 School of Chemical Engineering, Purdue University, West Lafayette, Indiana, United States of America; 3 Department of Physics, George Washington University, Washington, District of Columbia, United States of America; Schulze Center for Novel Therapeutics, Mayo Clinic, United States of America

## Abstract

Using a combination of small-angle X-ray scattering (SAXS) and fluorescence resonance energy transfer (FRET) measurements we have determined the role of the H3 and H4 histone tails, independently, in stabilizing the nucleosome DNA terminal ends from unwrapping from the nucleosome core. We have performed solution scattering experiments on recombinant wild-type, H3 and H4 tail-removed mutants and fit all scattering data with predictions from PDB models and compared these experiments to complementary DNA-end FRET experiments. Based on these combined SAXS and FRET studies, we find that while all nucleosomes exhibited DNA unwrapping, the extent of this unwrapping is increased for nucleosomes with the H3 tails removed but, surprisingly, decreased in nucleosomes with the H4 tails removed. Studies of salt concentration effects show a minimum amount of DNA unwrapping for all complexes around 50-100mM of monovalent ions. These data exhibit opposite roles for the positively-charged nucleosome tails, with the ability to decrease access (in the case of the H3 histone) or increase access (in the case of the H4 histone) to the DNA surrounding the nucleosome. In the range of salt concentrations studied (0-200mM KCl), the data point to the H4 tail-removed mutant at physiological (50-100mM) monovalent salt concentration as the mononucleosome with the least amount of DNA unwrapping.

## Introduction

In recent years, research investigating the problem of chromatin structure has increased dramatically. This is in a large part due to the important link between chromatin structure and epigenetics [1,2]. Early chromatin studies concentrated on the varying levels of density of genomic material within the nucleus which creates hetero- and euchromatin. This led to the discovery that a change between these densely-packed and less densely-packed phases could be brought about by changes in ion composition or in composition of the histone tails with the eventual conclusion that the physics of this process could largely be attributed to packaging of the nucleosome core particle [3].

One major focus of chromatin research since has concentrated on this DNA packing at the nucleosome level (see [4,5] for recent reviews). The canonical nucleosome consists of 147 base pairs of DNA wrapped around an octameric histone core of four proteins (H2A, H2B, H3, and H4). These proteins contain positively-charged, flexible tails that protrude from the core either through (in the case of H2B and H3) or around (in the case of H2A and H4) the DNA [6,7]. This structure, called the nucleosome core particle (NCP), is then connected to other NCPs through varying lengths of “linker” DNA. Driven by increasingly refined structures of the nucleosome [7–9] and nucleosome arrays [10], the question of the packing of nucleosomes into the 30nm fiber (or nucleosome condensation) and other higher-order structures has attracted some of the most active research and debate [11]. 

At the heart of these questions is the method through which cellular machinery gains access to the genetic content that is stored in the DNA around nucleosomes. Regardless of the state of packing of the nucleosome, this machinery must gain access to the DNA wrapped around the nucleosomes. Recent studies have shown that there are many possible mechanisms for accessing this DNA [4] including partial or complete nucleosome disassembly [12,13], nucleosome sliding [14], and partial DNA unwrapping [15–18]. 

The many studies on DNA unwrapping in the nucleosome show it to be a complex and highly tunable effect. Previous studies have shown that the process can be affected by DNA sequence [19–22] or methylation of the DNA [23,24], complete histone tail removal or acetylation [22,25,26] or removal or acetylation of both the H3 and H4 tails [26,27]. FRET studies have further explored the consequence of the acetylation of the H3 or H4 tails removed separately [28–30] and with H2A and H2B tails removed [31]. Similar studies have investigated the role of the surrounding buffer conditions [17,22,25,28,32] on DNA unwrapping. 

In this paper, we study the solution structure of recombinant nucleosome core particles in their wild-type form as well as with part of the H3 or H4 tails removed individually. We use a combination of SAXS, a global structure measurement, and DNA-end-labeled FRET, a local measurement, to determine the prevalence and extent of DNA unwrapping in all NCP constructs under a variety of ion conditions, and therefore infer the role of the histone tails and ion concentration in stabilizing the NCPs. These combined SAXS and FRET measurements demonstrate that while all NCPs measured exhibit DNA unwrapping, the extent of this unwrapping is strongly dependent on the histone tails of histones H3 and H4 and on the monovalent salt concentration. These data should be instrumental in understanding the physical processes behind the complex interplay between histone tail modifications and the various pathways through which cellular machinery gains access to genetic material.

## Methods

### Sample Preparation

Nucleosomes were prepared *in vitro* by mixing 147bp DNA fragments containing the Mouse Mammary Tumor Virus (MMTV) DNA sequence (see Materials S1 for sequence) and recombinant histone octamers. 

The 147bp MMTV DNA fragments were expressed in *E.coli* cells (TOP10) and purified as described by Dyer et. al. [33]. The fluorescently-labeled DNA fragments were prepared by PCR amplification using Fluorescein (FAM) and/or Tetramethylrhodamine (TAMRA) labeled primers (IDT DNA, Coralville, IA). DNA fragments labeled at their 5’ ends with FAM (donor-only label) or with FAM and TAMRA (donor-acceptor labeled) were prepared. The PCR products were purified by HPLC ion exchange (TSK-DEAE-5PW, Tosho Bioscience, King of Prussia, PA). The fluorescence labeling efficiency of TAMRA to the DNA was assessed by absorption spectroscopy and was found to be 99% [24]. 

Recombinant histone proteins H2A, H2B, H3 and H4 from *Xenopus laevis* were individually expressed in *E.coli* cells (BL21 cells) and purified by HPLC gel permeation chromatography (Sephacryl S-200HR, GE, Uppsala, Sweden) and HPLC ion exchange chromatography (Resource-S, GE, Uppsala, Sweden) as described by Luger et. al. [34]. In addition to the native histone proteins (referred to as wild-type or WT), we also expressed the H3 and H4 histone proteins truncated at their N-terminal ends (referred to as gH3 and gH4, respectively). The gH3 and gH4 histones were obtained by deleting the coding sequences of amino acids 1-27 for H3 and 1-10 for H4 by site-directed mutagenesis.

The histone octamers were refolded by mixing all four histone proteins, i.e. H2A, H2B, H3 (or gH3) and H4 (or gH4), at stoichiometric ratios and successive dialysis against refolding buffer (2M NaCl, 10mM Tris pH 7.5, 1mM EDTA and 5mM 2-mercaptoethanol). The refolded histone octamers, i.e. WT, gH3 and gH4, were purified by HPLC gel permeation chromatography (Superdex 200, GE, Uppsala, Sweden). The quality of the refolded histone octamers were verified using SDS-polyacrylamide gel electrophoresis (Figure S1 in Materials S1). 

Nucleosomes were reconstituted by mixing either fluorescently labeled DNA or unlabeled DNA with the refolded octamers using a KCl gradient (from 1.4M KCl to 10mM KCl). All NCP stocks were extensively dialyzed against TEK buffer (10 mM KCl, 10 mM Tris, 0.1 mM EDTA). The quality of the nucleosomes was assessed by 5% polyacrylamide gel (0.25xTBE buffer) as shown in Figure S2 in Materials S1. 

### FRET Measurements

#### Time-resolved lifetime spectroscopy

Fluorescent measurements and analysis were performed as described in [24]. Briefly, fluorescence decay curves of donor and donor-acceptor labeled nucleosomes were collected using a ChronosBH fluorescence lifetime spectrophotometer (ISS, Champlain, IL) with a 445nm, 20MHz laser pulse. Nucleosome concentration was kept above 1 μM (0.2 mg/mL) by titrating in unlabeled NCPs to prevent nucleosome dissociation [35]. An emission filter (505-545nm) was used to collect only the fluorescence emission from the donor dye. All emission spectra were collected at the magic angle (54.7°). Typical fluorescence decay curves from donor and donor-acceptor labeled samples are presented in Figures S7-9 in Materials S1.

All fluorescence decay curves were analyzed using VINCI Fluorescence Spectroscopy Analysis Software (ISS, Champlain, IL). The fluorescence intensity curves were fitted with a multi-exponential model $I(t)=\sum_{i=1}^{n}\alpha_{i}\exp(-t/\tau_{i})$ where *I*(*t*) is the fluorescence intensity at different times, α_*i*_, is the initial fluorescence intensity at t = 0, *n* is the number of fluorescent species and *τ*_*i*_ is the fluorescent lifetime (or decay time) of the *i* ^*th*^ species. The number of decay times used to fit the data was determined by the goodness of fit. The goodness of fit was evaluated using the chi squared value (χ^2^) and the residuals plot. The donor-only labeled samples were better fit with a one exponential model (n=1) while the donor-acceptor labeled samples were consistently better fit with a two exponential model (n=2). The fitting parameters and the goodness of fit of typical donor and donor-acceptor labeled samples are presented in [24] and Figures S7-9 in Materials S1.

 The average lifetime of FAM in the donor-acceptor labeled samples was calculated as the weighted average of the individual lifetimes, *τ*_*i*_, as follows:$\tau_{da,ave}=\sum_{i=1}^{n}\alpha_{i}\tau_{i}$. The average Energy Transfer Efficiency (*E*) was calculated using the average lifetime of the donor-acceptor labeled samples (τ_*da,ave*_) and the lifetime of the donor labeled samples (*τ*_*d*_) as follow$E=1-\tau_{da,ave}/\tau_{d}$, where $\tau_{d}$ is the lifetime of donor labeled nucleosome. The lifetime of donor labeled samples remained virtually unchanged in the studied salt concentration range, as shown in Figure S10 in Materials S1.

### SAXS Measurements

#### Data collection and processing

SAXS data were collected at the G-line station at the Cornell High Energy Synchrotron Source (CHESS). The beam had an energy of 9.97keV and a size of 250x250μm. Samples of [NCP] of approximately 12.5 μM (2.5 mg/ml) and 30μl were inserted into a capillary and oscillated to avoid beam damage. The same capillary was used for buffer and sample measurements to eliminate effects from the capillary. Aggregation was controlled through short exposures; signals were monitored for time-dependent aggregation. R_g_ profiles showed a linear Guinier region with R_g_ similar to those previously reported [19,27]. The samples were absolutely calibrated using scattering from a water sample and normalized by the radiation incident on the beam stop. Six to eight two-second exposures, each, for the buffers and samples were averaged and subtracted to create the final SAXS profile *I*(*q*), whereq=4πsin(θ)/λ, 2*θ* is the scattering angle, and λ is the x-ray wavelength. For sample-to-sample comparison, signals were matched at *q* = 0.04-0.08 Å^-1^ to control for slight differences in concentration; this region is known to be free of interparticle interference [36]. Analysis was done using RAW [37] and in-house MATLAB (The MathWorks, Natick, MA) functions.

#### CRYSOL modeling

All theoretical scattering work was done using the ATSAS package [38]. Theoretical scattering predictions and the fits to these predictions were performed using CRYSOL [39]. Data were fit from a *q* of 0.03-0.3 Å^-1^ with default options and background subtraction. Nucleosome models with fully-wrapped or partially unwrapped DNA were made for both extended and folded tails using Viewer Lite and Discovery Studio (Accelrys, San Diego, CA). DNA unwrapping of the nucleosomes was modeled by replacing a number of nucleosomal DNA (between 3 and 35 bp) with B-DNA of the same sequence. The amount of DNA unwrapping is reported by the number of basepairs that have been converted from nucleosome structure DNA to B-DNA. The effect of this removal on the scattering signal can be seen in Figure S4 in Materials S1. 

Tails were removed using a text editor; all structures were identical across constructs (WT, gH3, gH4) except for changes in respective tail lengths as indicated in the sample preparation section.

## Results

SAXS data were taken on all nucleosomes and the scattering data were compared to theoretical scattering predictions from CRYSOL on the 1kx5 crystal structure [7] (modified to remove tails where appropriate). To obtain the best fit to the data, multiple nucleosome models were made for each construct. The 1kx5 structure was first modified to have either extended or folded tails. Then structures were created with the DNA ends unmodified or with the DNA ends partially dissociated from the nucleosome. The nucleosome models contained from 0 to 35 basepairs of DNA converted from the reported crystal structure positions to those conforming to the canonical B-DNA structure. Finally, the tails were modified to match the measured construct (WT, gH3, or gH4). These structures were used to fit the measured scattering data and CRYSOL χ values were compared to assess the quality of the fit. 

**Figure 1:**
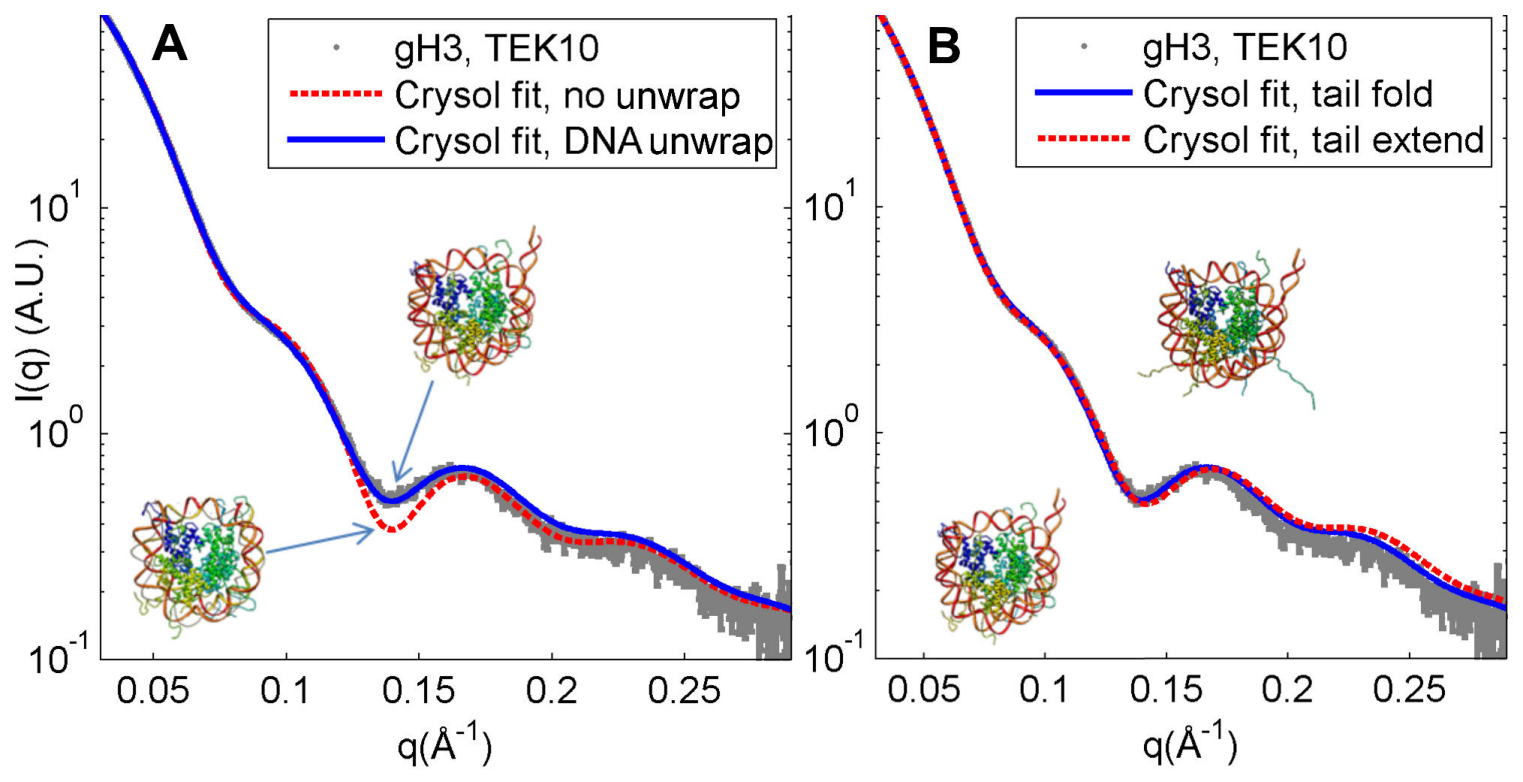
SAXS profile of gH3 nucleosome core particle at 10mM KCl compared to CRYSOL scattering predictions. In (*A*) we show the comparison between SAXS data for the gH3 nucleosome core particle (NCP) (dots) and CRYSOL predictions with folded tails and no DNA unwrapping (dashed, χ=7.6) or with ~20 bp of DNA unwrapped from one end of the nucleosome (solid, χ=4.7). The minima in the scattering prediction at approximately *q*=0.14 Å^-1^ for the NCP without unwrapping is deeper than the prediction for the NCP with unwrapping or the measured scattering profile. This shows that the data can only be described by the structure with the unwrapped DNA ends. In (*B*) we show the comparison between the CRYSOL predictions with DNA unwrapped and tails folded (solid, χ=4.7) or extended (dashed, χ=4.6). The differences between these signals are undetectable; both match the data (dots) comparably. Similarly, the differences between tail-extended and tail-folded models with the DNA fully wrapped are small (Figure S3 in Materials S1).

It was found that the scattering predictions from models without a partially-unwrapped DNA end could not be made to fit the x-ray scattering signal of any of the constructs. The gH3 construct is shown as it displays the largest deviation from crystal structures with the DNA completely wrapped (Figure 1). In particular, a local minimum was found in the scattering profile at *q*≈0.14 Å^-1^ that was not seen in the CRYSOL predictions of the scattering profile from the nucleosomes without unwrapped DNA. This feature has been seen in previous SAXS studies of nucleosome core particles and has been shown to be a consequence of DNA unwrapping [19,27]. In concordance with these studies, it was found that varying the amount of DNA unwrapping in the crystal structure caused the minimum to become shallower with more DNA unwrapping and to become deeper with less unwrapping (with the deepest minimum occurring in the original, fully-wrapped crystal structure). The best fit to the data was found by unwrapping ~20bp of one end of the DNA from the nucleosome (Figure 1A). Theoretical scattering plots from structures with both DNA ends unwrapped consistently showed worse or equivalent fits to the SAXS data. This result is in agreement with pulling experiments showing differential binding of non-palindromic DNA sequences to the nucleosome [40]. While it is possible to observe the effects of DNA dissociation through R_g_ or D_max_ analysis, these analyses are both measurements of the total dimensions of the particle and therefore could possibly be reporting on either tail extension or DNA unwrapping [25,27]. In contrast, we find that extending or folding the histone tails produced minimal differences when fitting the data to CRYSOL predictions to the minimum at *q*=0.14 Å^-1^ (Figures 1B; S3, and S11 in Materials S1), confirming that this minimum at *q*=0.14 Å^-1^ is a specific reporter on the amount of DNA unwrapping in the nucleosome. This allows us to use the depth of this minimum to determine the extent of DNA unwrapping in nucleosomes: The deeper the minimum, the closer the nucleosome’s conformation is to the fully-wrapped DNA crystal structure.

**Figure 2:**
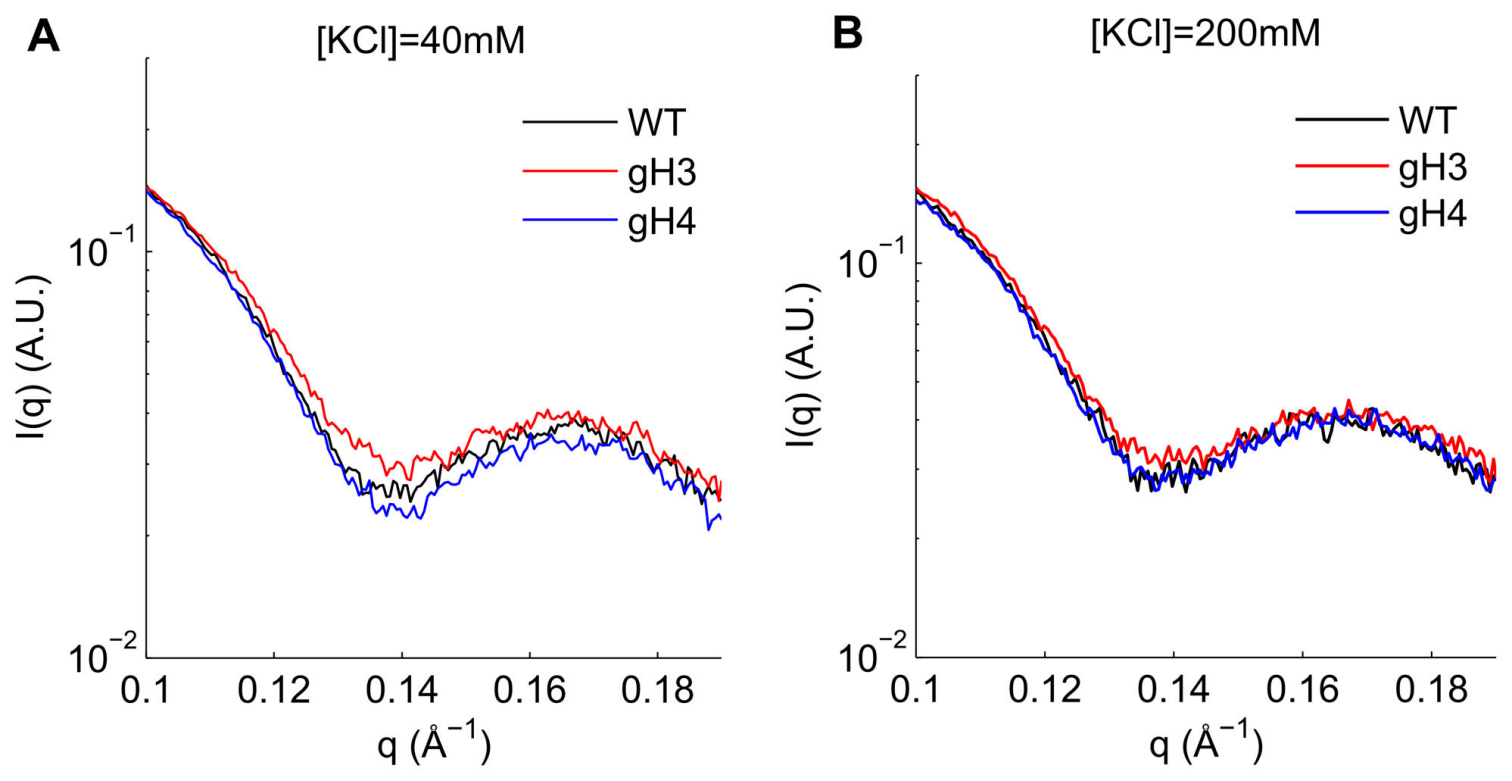
SAXS profiles of all wild-type, gH3, and gH4 nucleosomes at two salt concentrations. SAXS signals of the wild-type (black), gH3 (red), and gH4 (blue) constructs in 40 mM (*A*) and 200 mM (*B*) KCl are shown. The lower minima at *q*=0.14 Å^-1^ indicates less DNA unwrapping (see Figure 1). Note that in the 40 mM KCl signal, the signals are arranged in order of increasing DNA unwrapping from gH4 to WT to gH3; however the differences between gH4 and WT are minimal. For the 200 mM KCl SAXS data, the WT and gH4 constructs are indistinguishable at *q*=0.14 Å^-1^ within the noise of the SAXS signal, while the gH3 shows significant DNA unwrapping in comparison. These results were independent of signal matching region (Figure S5 in Materials S1).

Using this reporter of DNA unwrapping, we looked at wild-type nucleosomes and gH3 and gH4 mutants under lower salt (40 mM KCl) and higher salt (200 mM KCl) conditions. This analysis is shown in Figure 2. Two effects are immediately apparent. First, the amount of DNA unwrapping is different for each NCP construct. At 40 mM KCl, the minimum of the gH3 nucleosome is significantly shallower than the recombinant wild-type (WT). This indicates that the gH3 nucleosome shows greater DNA unwrapping when compared to the wild-type. This effect has been shown in other work [28,29]. This enhanced DNA unwrapping compared to the wild-type due to the removal of the H3 tails is also seen in the 200 mM KCl SAXS data, again through a shallower minimum at *q*=0.14 Å^-1^.

At 40 mM KCl the minimum at *q*=0.14 Å^-1^ is slightly deeper for the gH4 complex when compared to the wild-type. While this could indicate that the DNA in the gH4 nucleosome exhibits reduced unwrapping compared to the wild-type, the difference is minimal; a more detailed analysis of the SAXS data at this salt concentration (below) was unable to confirm this difference. At the higher salt concentration of 200 mM KCl, the gH4 and wild-type signals are identical, indicating that the amount of unwrapping in these complexes is indistinguishable by SAXS. 

The SAXS data has additional information contained in the regions outside of the *q*=0.14 Å^-1^ region. The data were further analyzed by fitting constructs with different amounts of DNA unwrapping to the SAXS data (Table 1; S4 and S6 in Materials S1). The observations confirm much of what is seen in the above analysis. At [KCl]=40 mM, the WT and gH4 constructs show minimal amounts of DNA unwrapping (~10bp) while the gH3 data is best fit by a construct with 25bp of one DNA end unwrapped (Table 1). When the salt concentration is increased to [KCl]=200 mM, the gH3 and gH4 data are still best fit by the 25pb and 10bp models, respectively, while the WT data is now better fit by a 20bp unwrapped model. 

**Table 1:** Test of different amounts of DNA unwrapping for each NCP construct.

	**[KCl]=40 mM**		**[KCl]=200 mM**	
**Construct**	**bp Unwrap**	**χ^2^**	**bp Unwrap**	**χ^2^**
WT	10	3.5	20	2.7
gH4	10	4.6	10	3.3
gH3	25	4.7	25	4.8

Number of basepairs (bp) unwrapped in theoretical models that best fit SAXS data using CRYSOL predictions and corresponding chi-squared values. The gH3 construct is always significantly more unwrapped, while the wild-type and gH4 constructs are only distinguishable at higher salt concentrations. It should be noted that at the high salt concentrations the gH4 construct fit a 20 basepair unwrapped model almost as well as the 10 basepair model (see Materials S1).

It should be noted, however, that there is some indication in the data that the fits at [KCl]=200 mM point to a combination of states, especially in the case of the gH4 and WT constructs (Figure S6 in Materials S1). The CRYSOL fits for these data show two minima in the χ^2^ values as a function of number of basepairs unwrapped. One minimum is for the 10bp unwrapping (as seen in the 40 mM data) while the second minimum in both the gH4 and WT constructs is seen at 20bp unwrapping. The data suggests that both gH4 and WT SAXS profiles contain signatures that are indicative of both 10bp and 20bp unwrapping and that the features of the 20bp unwrapping dominate the CRYSOL fit in the WT data.

**Figure 3:**
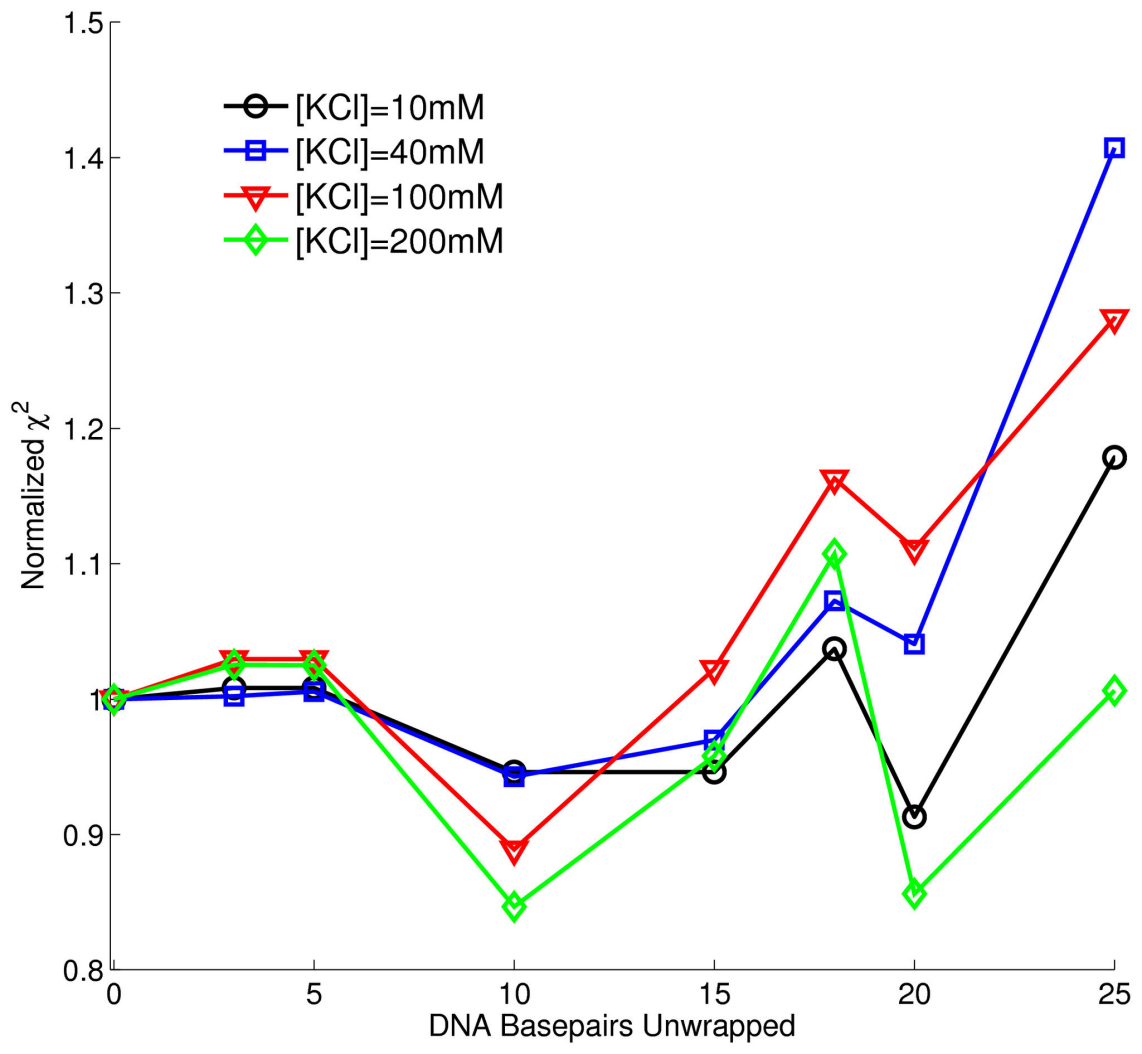
Goodness of fit between gH4 data at multiple salt concentrations and CRYSOL predictions of DNA-unwrapped nucleosomes. Chi-squared value (goodness of fit) between CRYSOL predicted scattering and SAXS data for the gH4 construct at [KCl]=10mM (circles), 40mM (squares), 100mM (triangles), and 200mM (diamons) with various amounts of DNA basepairs unwrapped. For all salt concentrations, there appears to be two local minima in χ^2^, one at 10bp unwrapped and one at 20bp unwrapped. The global minimum (best fit) changes from the 20bp unwrapped construct at [KCl]=10mM to the 10bp unwrapped construct at [KCl]=40mM and 100mM. At [KCl]=200mM, there is again a strong shift towards the 10bp unwrapped construct; the 10bp and 20bp χ^2^ minimum are approximately the same.

The gH4 complex was further studied to determine the conformation with the least amount of DNA unwrapping (Figure 3). As in the studies of the various constructs, it was found that there were two local minima of χ^2^ (a maximum in goodness of fit) at all salt concentrations; one existed at ~10bp unwrapped, the other at ~20bp. The data is best fit (has a global minimum in χ^2^) by the 20bp unwrapped construct at 10mM KCl but is best fit to the 10bp unwrapped structure at 40mM and 100mM KCl. At 200mM KCl, the data is equally well described by the 10bp and 20bp constructs.

To complement these SAXS data investigating the DNA unwrapping, DNA end-labeled FRET was used. FRET labels were attached to both DNA ends and the FRET efficiencies were measured. The energy transfer efficiency increases as the DNA ends move more closely together and decreases as the DNA ends separate. The FRET measurements of the WT, gH3, and gH4 complexes were measured as a function of monovalent salt (KCl) from 10mM to 230mM. 

**Figure 4:**
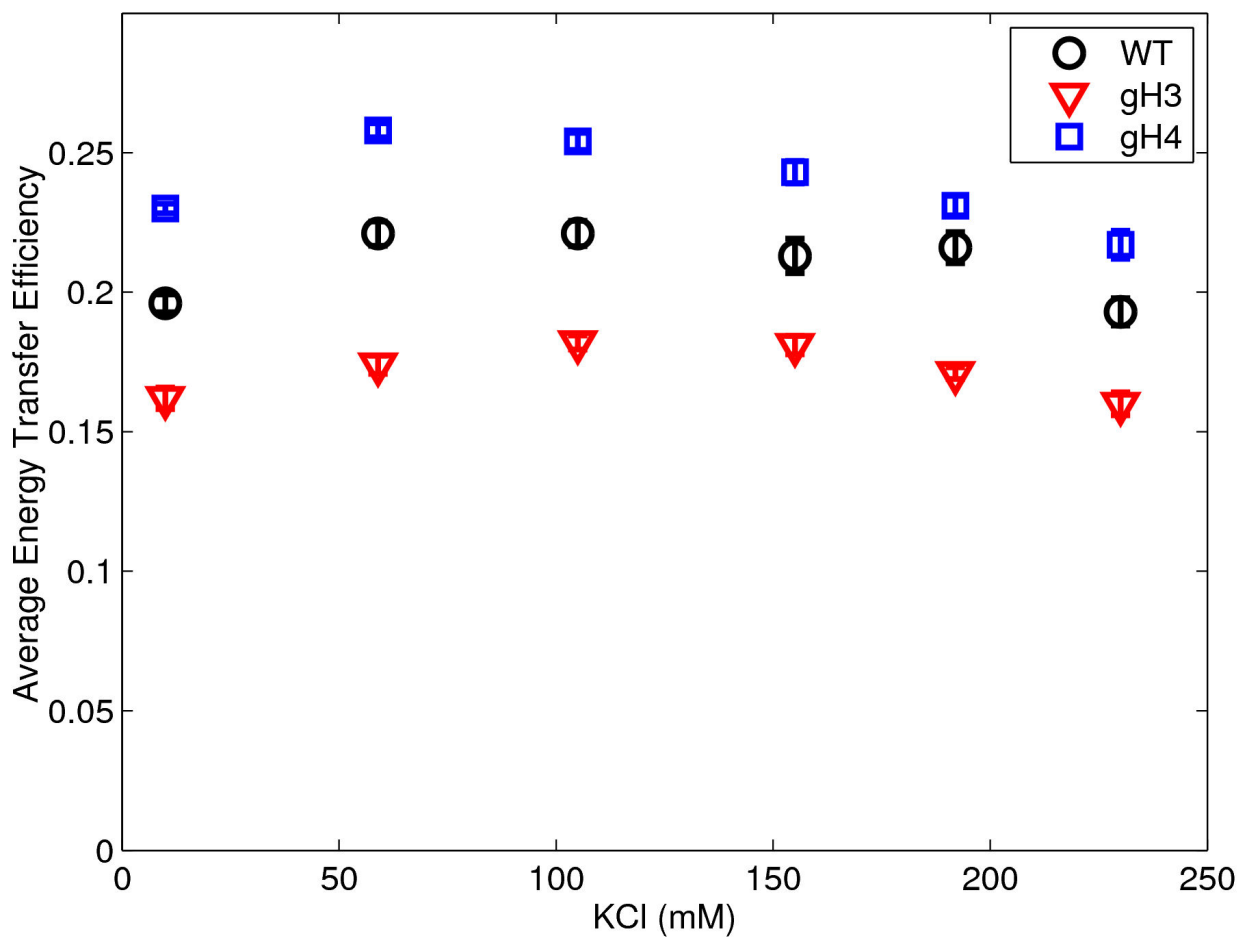
Plot of average energy transfer efficiency between the two end-labeled DNA in the nucleosome versus salt concentration. Plot of average energy transfer efficiency (or FRET signal) between the two fluorescently labeled DNA ends in the nucleosome versus salt concentration for the wild-type (circles), gH3 (squares), and gH4 (triangles) constructs; the standard error is plotted and is seen to be approximately the size of the symbols. A larger transfer efficiency indicates a shorter distance between the DNA ends. The data show a minimum distance (maximum FRET) for all constructs between 50 and 100mM KCl indicating a minimum in the DNA unwrapping in this vicinity. The data also show an increase in end-to-end DNA distance in the gH3 nucleosomes (decreased FRET) compared to the wild-type indicating great DNA unwrapping with the removal of the H3 tails. The gH4 nucleosomes, meanwhile, shows decreased end-to-end DNA distance (increased FRET) compared to the wild-type nucleosomes, indicating a stabilization of the nucleosome through removal of the H4 tails. These data agree well with the SAXS analyses (within error of the larger SAXS uncertainties) in Figure 2 and Table 1.

As is seen in Figure 4, there is initially a slight increase in all signals as we increase the monovalent salt from 10mM to 100mM. This corresponds to the DNA ends getting closer together and is interpreted as the DNA ends adhering more closely to the histone core. As the salt was increased past ~100mM, this trend is reversed as the FRET signal decreases indicating an increase in the unwrapping of the DNA ends. These results point to a minimum in DNA unwrapping (or a maximum of nucleosome stability) between 50mM and 100mM for all constructs. The closer adherence of the DNA ends when approaching 100mM has been reported previously in [28], but this study was unable to investigate past 80mM monovalent ion due to the dissociation of the nucleosomes attributed to the low concentration of their experiments.

A second feature of Figure 4 is the relative amounts of unwrapping of the various nucleosome mutants. Similar to the SAXS data, the gH3 shows consistently more unwrapping than the WT or gH4 complex. 

Also as in the SAXS data, the gH4 complex shows less DNA unwrapping than the wild-type. However, the increased sensitivity of the FRET technique shows that the gH4 complex exhibits less DNA unwrapping at all KCl concentrations explored in this study. Of all of the constructs and salt concentrations studied, it was found that the gH4 construct at approximately 50-100mM [KCl] represented the nucleosome with the least amount of DNA unwrapping.

## Discussion

The combination of the two techniques used in this study can give us important complementary structural information. FRET is a local structure technique that reports on the relative distance between the two FRET labels. When combined with the SAXS measurements that report global structure, we can correlate the measured structural changes at local and global scales and provide an accurate measure of the conformational change of a mononucleosome, and in this case quantify the effects of DNA unwrapping.

The strengths of each technique can be seen in the above results. FRET, with its strong sensitivity to inter-dye distances, can easily distinguish the DNA unwrapping of the various constructs from each other and monitor the subtle effects of changes in salt concentration. The SAXS data, while less sensitive, can give more detailed structural information. In the case of this data, it reveals that the data seems to be revealing two distinct populations of nucleosome (of ~10bp and ~20bp DNA unwrapped nucleosomes) and that the change in the relative amounts of these populations seems to be what determines the resulting apparent total unwrapping. This is consistent with previous work done by the authors investigating the ratio of FRET populations in time-resolved experiments [24].

The most striking result from these combined measurements are found in Figures 2 and 4 and Table 1. While the removal of the H3 tail causes a drastic increase in the measured DNA unwrapping of the NCP complex, the removal of the H4 tail has no effect on the SAXS data and actually shows a decrease in the unwrapping of the DNA in the FRET data. Interestingly, Bertin et al. performed SAXS studies on nucleosomes that had both the H3 and H4 tails removed [27] as well as all tails removed [25]. They found in both studies that the removal of tails led the nucleosomes to adopt a more open form, with the DNA ends detached from the nucleosome core. This seems to indicate that the DNA unwrapping effect of the H3 tail removal is dominant over the effects of the removal of the H4 tail.

There are two likely mechanisms for the destabilization of the NCP complex due to the H3 tail removal. These include steric hindrance of the tail and electrostatic effects. The former would need to include a mechanism where the H3 tail, which protrudes between the DNA, acts as a “close pin” to somehow trap the DNA against the histone. Recent theoretical work has pointed to the possibility of steric effects in H3 tail-DNA interactions [41], however their work suggested a destabilizing effect of the H3 tail, exactly the opposite as seen in our data. The agreement between our results and previous experiments on the acetylation of the H3 tails [28] (rather than this work’s truncating of the tails), seems to indicate an electrostatic rather than steric effect.

The more intriguing result is the stabilization of the DNA ends when the H4 tails are removed as seen through the FRET experiments. This indicates a direct or indirect destabilizing interaction between the H4 tail and the nucleosomal DNA. A similar DNA-stabilizing effect for acetylated H4 tails (rather than tail-removed H4 histones) previously measured seems to indicate an electrostatic mechanism [28]. However, the acetylated H4 tails only showed this effect for long linker DNA lengths (DNA length >223 bp). The interpretation of the mechanism is further complicated by the shortening effect on the H4 tails due to increased alpha helicity after acetylation and the lower nucleosome concentrations used for these experiments [42]. It is known that the H4 tail interacts with an acidic patch in histone H2A which seems to have effects on the DNA unwrapping by modifying direct H2A-DNA interactions [43], although whether our slight shortening of the H4 tail would enhance or diminish this interaction is unclear. Direct, destabilizing contacts between the H4 tails and DNA can also not be ruled out. Finally, the fact that this stabilizing effect is seen only in the FRET measurements could either be a difference in sensitivities of these techniques, or indicate a difference in the dynamic behavior of the gH4 and WT complexes that is measurable by FRET but absent in bulk measurements like x-ray scattering. Further experiments looking at various amounts of H4 shortening and DNA linker lengths are needed to clarify the mechanism through which the shortening of the H4 tail might induce this more stable DNA wrapping. 

Finally, the DNA positioning sequence used cannot be ignored. Much of the previous work looking at the effects of tail removal on nucleosome stability and DNA unwrapping has concentrated on the strongly positioning 601 sequence. Nucleosomes made with this strongly binding DNA sequence are expected to show reduced unwrapping when compared to more loosely binding biological sequences, as has been shown in studies that compare the DNA unwrapping around nucleosomes for various sequences of DNA [19,20,22,44]. Future experiments that investigate DNA sequence and H3 or H4 tail removal simultaneously should be interesting in separating the effect of these two strong contributors to the DNA wrapping of the nucleosome core particle.

In conclusion, we have measured the SAXS profiles for recombinant wild-type, gH3, and gH4 nucleosome core particles. We found that these data can only be described by the unwrapping of the DNA ends and that the data are likely reporting on two distinct populations of DNA unwrapped nucleosomes of approximately 10 and 20 bp of unwrapping. Furthermore, we found, through the SAXS measurements combined with FRET measurements, that the removal of the H3 tails destabilizes the core particle, causing increased DNA unwrapping, while the removal of the H4 tails likely has the opposite effect and actually causes the DNA to become more strongly associated with the histone octamer. While these differences remained regardless of monovalent salt concentration, we found that the DNA end unwrapping could be suppressed or enhanced by changes in monovalent salt with a maximum in the DNA wrapping of the NCP at ~50-100mM KCl. As changes to the wrapping of the DNA around the nucleosome by histone tails is intimately related to the accessibility of genetic information, these findings should be vital in determining the role of nucleosomes in epigenetic regulation of the cell. 

## Supporting Information

Materials S1 **The supplementary materials file contains the DNA sequence used and 11 supplementary figures as mentioned in the text.**(DOC)
