# Supplementary material for: Solution Scattering and FRET Studies on Nucleosomes Reveal DNA Unwrapping Effects of H3 and H4 Tail Removal
Source: PLoS One. 2013 Nov 12;8(11):e78587. doi: 10.1371/journal.pone.0078587 (PMC3827064; doi:10.1371/journal.pone.0078587)
Supplement: Materials S1 — The supplementary materials file contains the DNA sequence used and 11 supplementary figures as mentioned in the text.(DOC) [file pone.0078587.s001.doc]

# Supplementary Materials

Exact DNA sequence used in recombinant nucleosomes:

ACTTGCAACAGTCCTAACATTCACCTCTTGTGTGTTTGTGTCTGTTCGCCATCCCGTCTCCGCTCGTCACTTATCCTTCACTTTCCAGAGGGTCCCCCCGCACACCCCGGCGACCCTCAGGTCGGCCGACTGCGGCACAGTTTTTTG

## Supplementary Figures:

**Figure S1:** SDS-PAGE of recombinant NCP histones (“intact” refers to the wild-type histones with all histone tails).

**
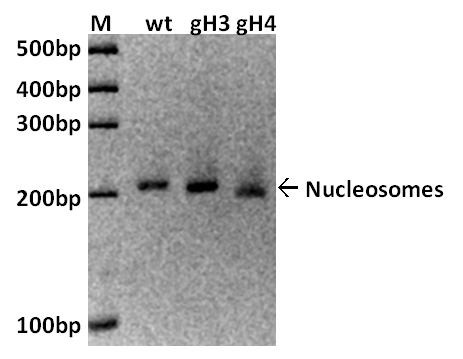
**

**Figure S2:** Typical 5% polyacrylamide gel of reconstituted nucleosomes, stained with EtBr.


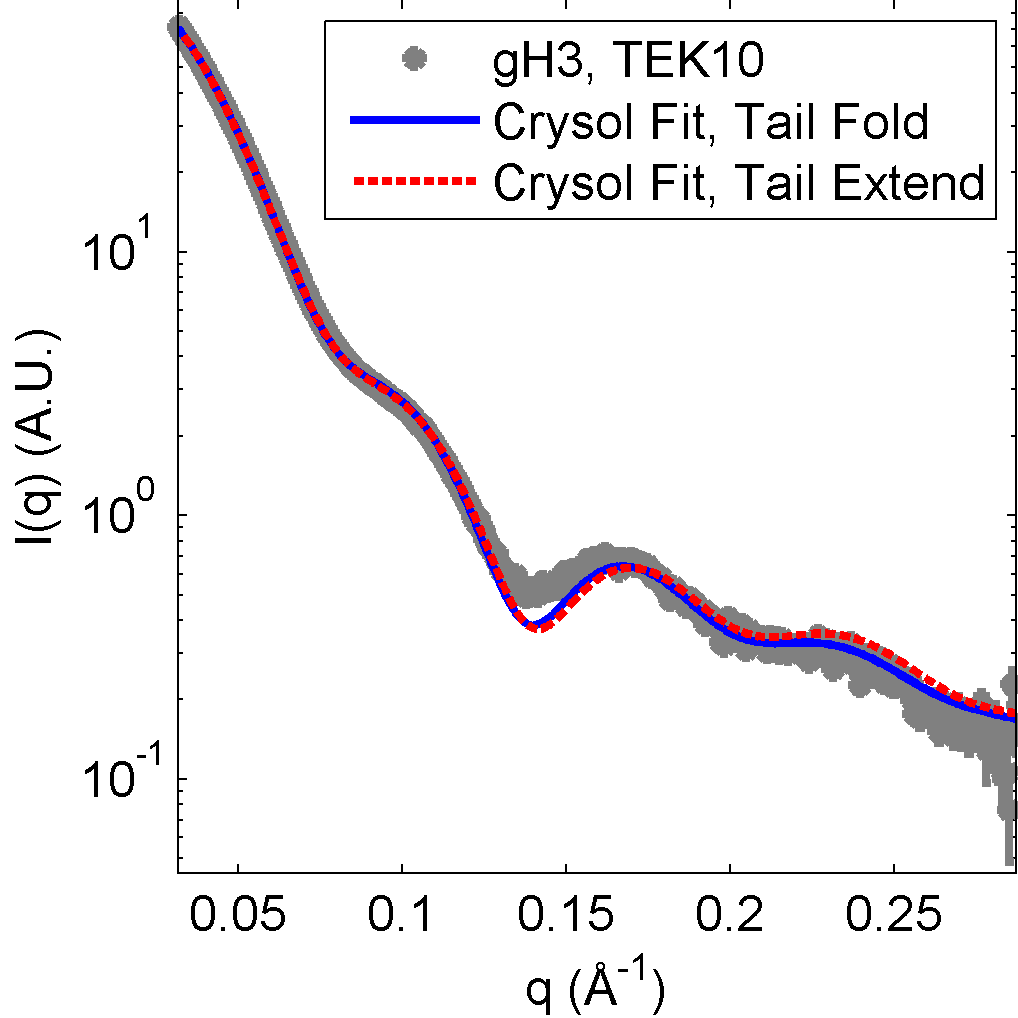


**Figure S3:** SAXS profile of gH3 nucleosome core particle at 10mM KCl compared to CRYSOL scattering predictions with DNA ends fully wrapped but with tails either folded (solid, χ=7.9) or extended (dashed, χ=7.1). While the extended tails fit slightly better (albeit not in the q=0.13Å^-1^ region of interest), the differences between these signals are much smaller than the changes from DNA unwrapping (see main text).

It should be noted that while we have modeled the histone tails in the PDB structures as perfectly extended, it is very unlikely that they will be in this configuration. The tails are known to be very flexible and are likely in a random walk configuration if extended. We have simulated a structure with the tails near maximum extension to try to maximize their effect on the scattering, but still see little difference in the scattering. This same discussion applies to figure S11.


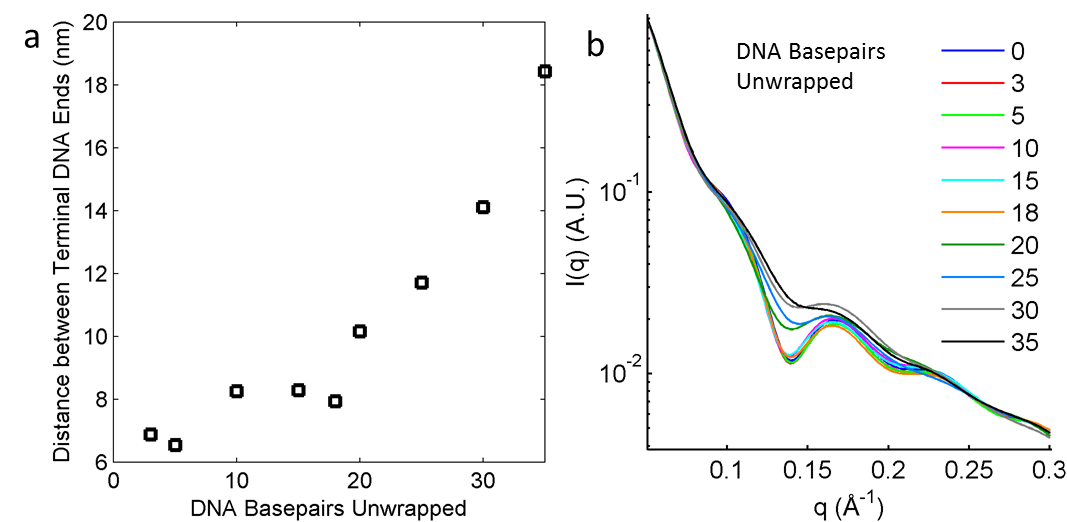


**Figure S4:** a) Plot showing the DNA end-to-end distance for the theoretical constructs created with increasing amounts of DNA basepairs unwrapped from the nucleosome. Models were created by replacing the nucleosomal DNA with B-DNA of the same sequence. b) Plot showing the variation of the scattering signal predicted by Crysol as the number of DNA basepairs unwrapped is increased. The signals are matched at q=0.04-0.06Å^-1^ to match the data analysis. As can be seen, the major change at q=0.13Å^-1^ occurs when approximately 20 basepairs are unwrapped from the nucleosome and increases slightly as this number is increased to 35.

**
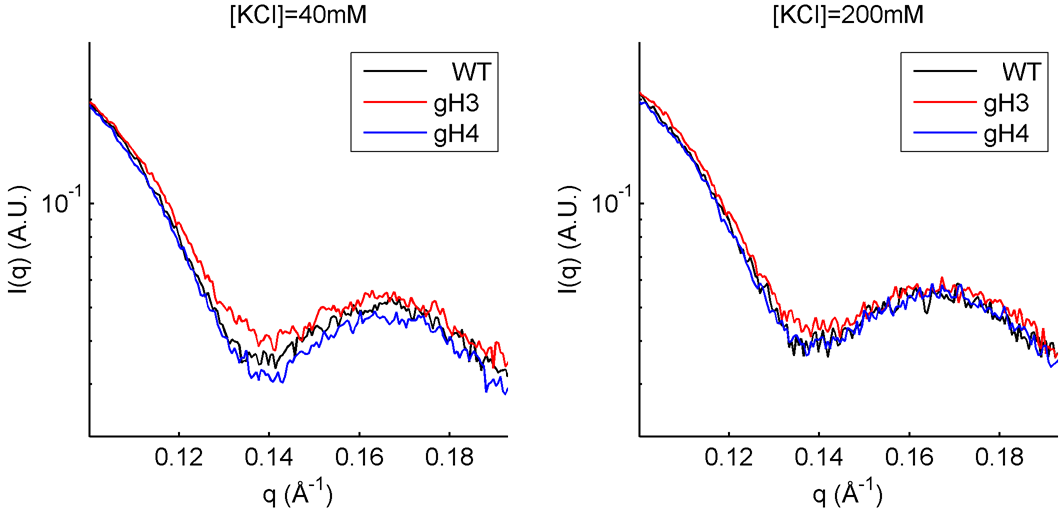
**

**Figure S5:** Plot of data from Figure 2 with alternative concentration matching region (data matched at q=0.04-0.1 Å^-1^) showing independence of results on sample concentration correction. As in Figure 2, the results show noticeable differences in all three constructs in DNA unwrapping at [KCl]=40mM (with unwrapping amount following the order gH3>WT≈gH4). At [KCl]=200mM, the gH4 and WT signals are indistinguishable in DNA unwrapping (gH3>WT≈gH4).


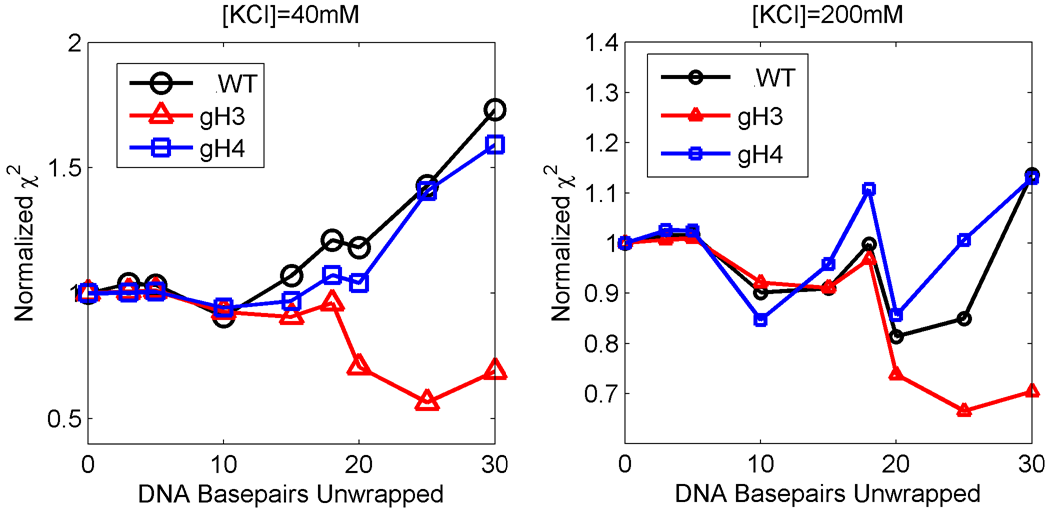


**Figure S6:** Chi-squared value (goodness of fit) between Crysol predicted scattering and SAXS data for constructs with various amounts of DNA basepairs unwrapped. At [KCl]=40mM, a minimum is found with 10bp unwrapped for the WT and gH4 constructs, while gH3 has a minimum at 25bp unwrapped. At [KCl]=200mM, the gH3 minimum remains unchanged while a new minimum (local for gH4, global for WT) appears at 20bp for the WT and gH4 constructs. The global minimum for gH4 is at 10bp. The two minima may indicate a distribution of states in these constructs at this salt concentration.

| **a**  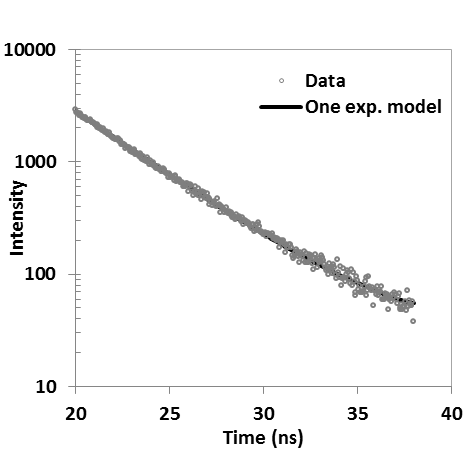 | **b**  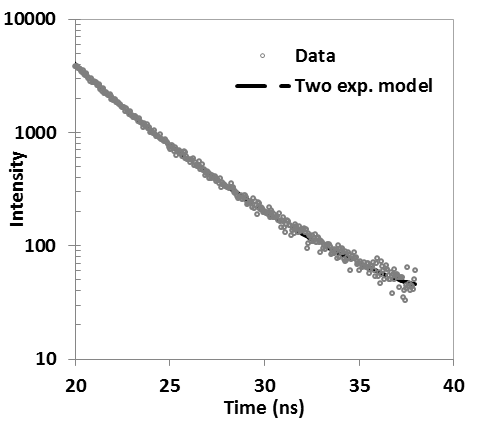 |
| --- | --- |
| 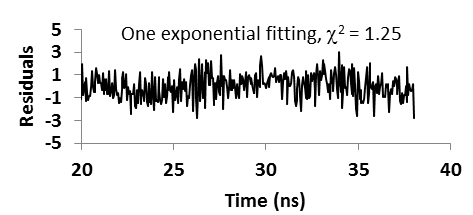 | 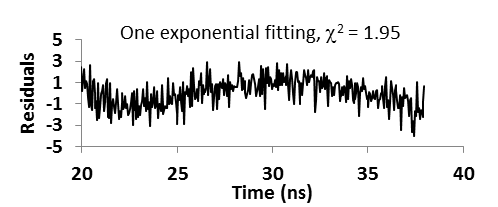 |
|  | 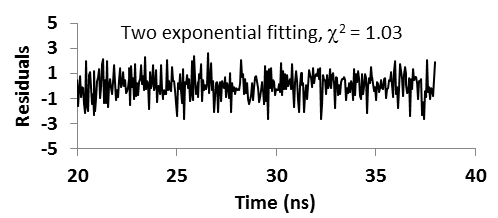 |

**Figure S7.** Typical fluorescence decay curves of wild type nucleosomes. a) Donor-only labeled nucleosomes. The fluorescence decay of the donor-only labeled sample was fit with a one exponential model. The fit gives a single lifetime τ = 3.76ns with a χ^2^ =1.25. b) Donor-acceptor labeled nucleosomes. The fluorescence decay of the donor-acceptor labeled sample was fitted with a one exponential and a two exponential model (χ^2^ of 1.95 and 1.03 respectively). The two exponential model fit gives two lifetimes τ_1_ = 1.75ns and τ_2_ = 3.69ns and a fraction of the fast lifetime (*f_1_*) at 0.359.

| **a**  **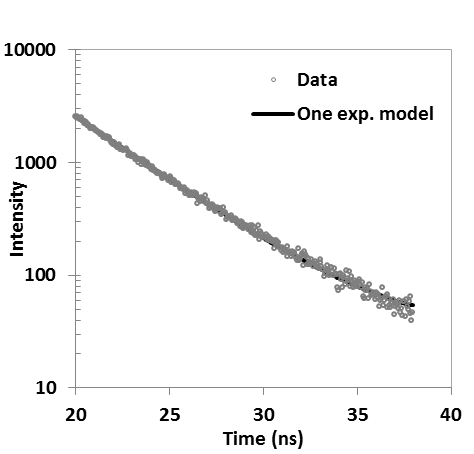** | **b**  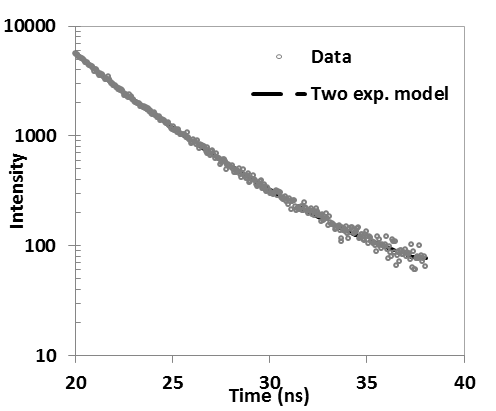 |
| --- | --- |
| 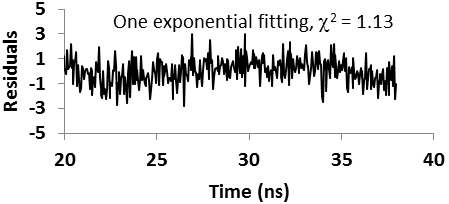 | 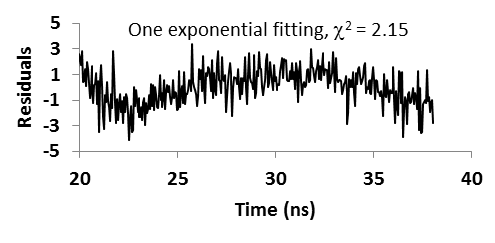 |
|  | 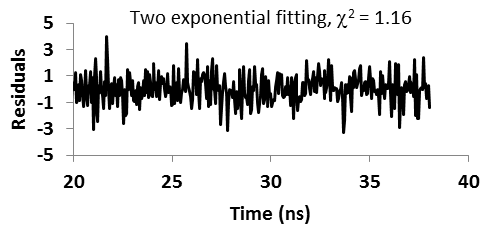 |

**Figure S8.** Typical fluorescence decay curves of gH3 nucleosomes. a) Donor-only labeled nucleosomes. The fluorescence decay of the donor-only labeled sample was fit with a one exponential model. The fit gives a single lifetime  = 3.77ns with a χ^2^ =1.13. b) Donor-acceptor labeled nucleosomes. The fluorescence decay of the donor-acceptor labeled sample was fit with a one exponential and a two exponential model (χ^2^ of 2.15 and 1.16 respectively). The two exponential model fit gives two lifetimes τ_1_ = 1.81ns and τ_2_ = 3.76ns and a fraction of the fast lifetime (*f_1_*) at 0.325.

| **a**  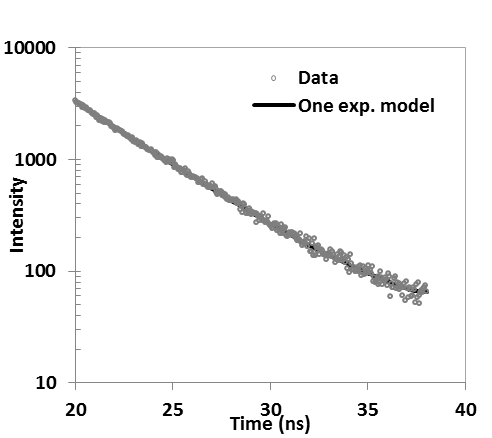 | **b**  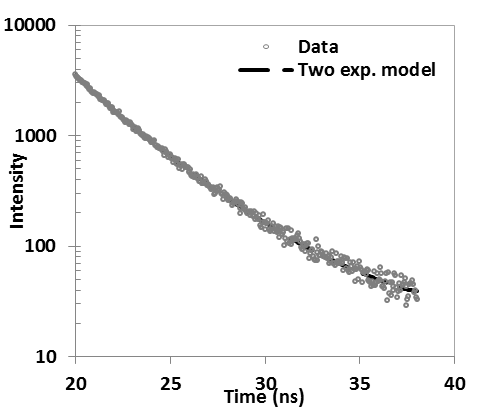 |
| --- | --- |
| 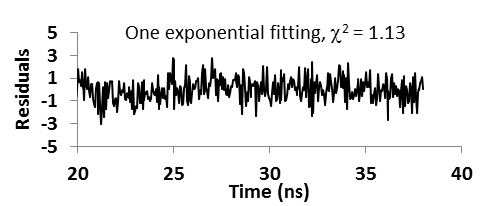 | 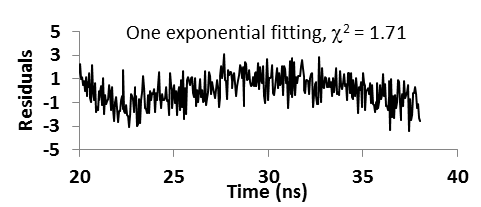 |
|  | 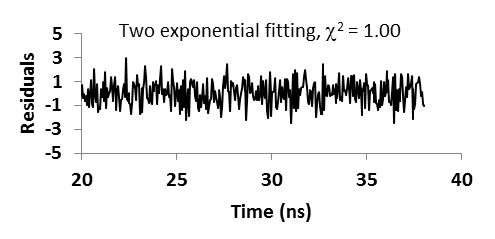 |

**Figure S9.** Typical fluorescence decay curves of gH4 nucleosomes. a) Donor-only labeled nucleosomes. The fluorescence decay of the donor-only labeled sample was fit with a one exponential model. The fit gives a single lifetime τ = 3.77ns with a χ^2^ =1.13. b) Donor-acceptor labeled nucleosomes. The fluorescence decay of the donor-acceptor labeled sample was fit with a one exponential and a two exponential model (χ^2^ of 1.71 and 1.00 respectively). The two exponential model fit gives two lifetimes τ_1_ = 1.79ns and τ_2_ = 3.57ns and a fraction of the fast lifetime (*f_1_*) at 0.374.


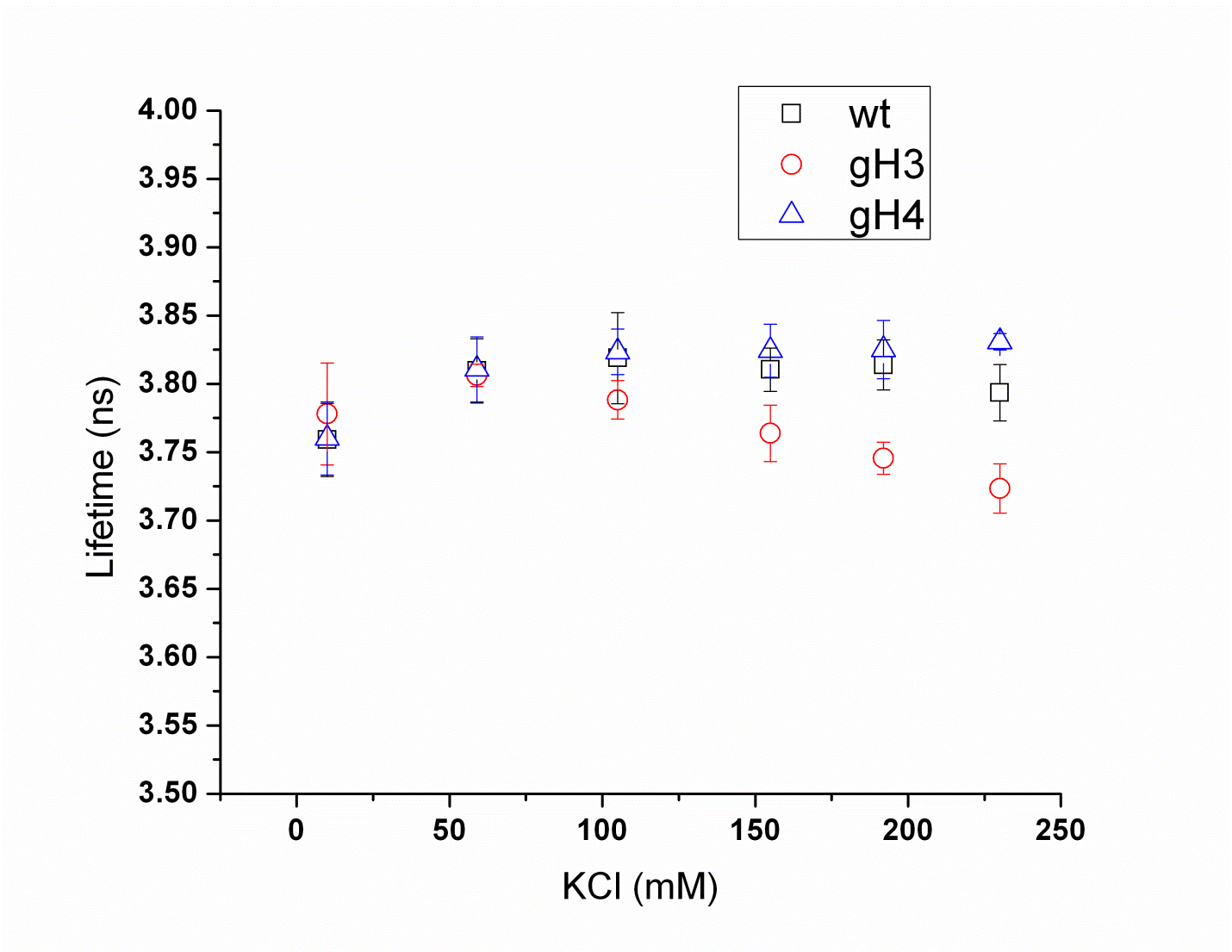


**Figure S10.** Dependence of donor-labeled nucleosomes lifetime on monovalent salt concentration. The FRET efficiencies were calculated using the measured donor lifetime at the same KCl concentrations as the dual labeled lifetimes. Therefore, we expect to have accounted for the salt effects in calculating FRET efficiencies. The donor lifetime does not exhibit a particularly strong dependence on salt concentrations, increasing in KCl seems to increase donor lifetime for all constructs initially, suggesting the potential release of DNA end fragments from histone octamer surface.


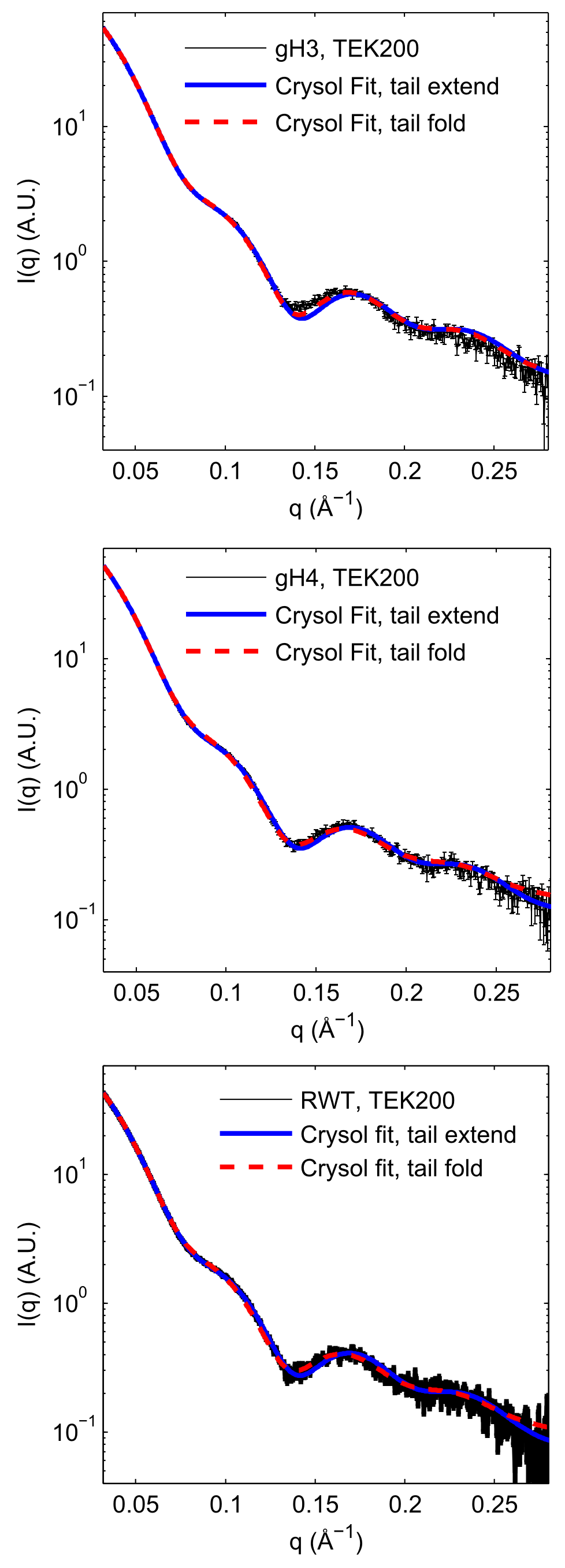


**a )**

**b**

**c**

**Figure S11.** Comparison of CRYSOL predictions of PDB structures with extended (solid) and folded (dashed) tails to a) gH3, b) gH4, and c) WT 200mM KCl scattering patterns. In all PDB structures, there was the same amount of DNA unwrapping (~20bp from one side) in the PDB structure. There is almost no difference in the quality of the fit to structures with extended or folded tails.
